# The experience of hospital care for older surgical patients and their carers: A mixed‐methods study

**DOI:** 10.1111/ajag.13176

**Published:** 2023-02-27

**Authors:** Janani Thillainadesan, Helen Box, Leanne Kearney, Vasi Naganathan, Michelle Cunich, Sarah J. Aitken, Sue R. Monaro

**Affiliations:** ^1^ Department of Geriatric Medicine Concord Hospital Sydney New South Wales Australia; ^2^ Faculty of Medicine and Health The University of Sydney Camperdown, Sydney New South Wales Australia; ^3^ Centre for Education and Research on Ageing Concord Hospital Sydney New South Wales Australia; ^4^ Sydney Health Economics Collaborative Sydney Local Health District Camperdown, Sydney New South Wales Australia; ^5^ Concord Institute of Academic Surgery, Concord Hospital Sydney New South Wales Australia; ^6^ Department of Vascular Surgery Concord Hospital Sydney New South Wales Australia; ^7^ Susan Wakil School of Nursing The University of Sydney Sydney New South Wales Australia

**Keywords:** aged, caregiversfrailty, geriatrics, health services, surgery

## Abstract

**Objective:**

A growing proportion of older adults are undergoing surgery, but there is a paucity of patient and carer experience research in this group. This study investigated the experience of hospital care in an older vascular surgery population for patients and their carers.

**Methods:**

This was a mixed‐methods convergent design, including simultaneous collection of quantitative and qualitative research strands by combining open‐ended questions with rating scales in a questionnaire. Recently hospitalised vascular surgery patients aged ≥65 years at a major teaching hospital were recruited. Carers were also approached to participate.

**Results:**

Forty‐seven patients (mean age 77 years, 77% male, 20% with a Clinical Frailty Scale score >4) and nine carers participated. The majority of patients reported that their views were listened to (*n* = 42, 89%), they were kept informed (*n* = 39, 83%), and were asked about their pain (*n* = 37, 79%). Among carers, seven reported their views were listened to and that they were kept informed. Thematic analysis of patients' and carers' responses to open‐ended questions about their experience of hospital care revealed four themes in terms of what mattered to them: fundamental care including hygiene and nutrition, comfort of the hospital environment such as sleep and meals, being informed and involved in health‐care decision‐making, and treating pain and deconditioning to help recovery.

**Conclusions:**

Older adults admitted to hospital for vascular surgery and their carers, valued highly the care that met both their fundamental needs and facilitated shared decisions for care and recovery. These priorities can be addressed through Age‐Friendly Health System initiatives.

**Practice Impact**

The study reveals that older surgical patients and their carers seek hospital care that goes beyond just a successful operation. Such care requires a collaborative multidisciplinary approach with a patient‐centerd focus on fundamental care and recovery.

## 1 INTRODUCTION

Despite being disproportionately affected by disease and treatment‐related complications, old and frail patients are underrepresented in clinical studies.^1^ The paucity of research involving this patient group applies particularly to patient‐reported outcomes and experiences of older patients undergoing surgery, even though they are a growing proportion of the surgical population.^2^, ^3^, ^4^ An ageing population, combined with greater uptake of minimally invasive endovascular techniques, has meant that vascular surgeons can offer interventions to older patients. It is important to understand what matters to older surgical patients as their health priorities differ from those of younger patients.^5^

Patients' perceptions of health‐care outcomes and their experience of receiving care can be measured using patient‐reported outcome measures (PROMs) and patient‐reported experience measures (PREMs), respectively.^6^ To date, studies on older vascular surgery patients have infrequently investigated PROMs, particularly functional status and quality of life.^7^ A systematic review of the outcomes reported in abdominal aortic aneurysm studies in patients aged ≥80 years found only five of 40 studies reported on patient‐centred outcomes; two reported on discharge destination, and three reported on quality of life.^7^ Furthermore, there have been limited studies examining the experience of hospital care in older vascular surgery patients.^8^ Also important is understanding how the environment and broader contextual factors influence the patient's care encounter in hospital.^9^

An increasing number of older Australians have informal carers, yet service providers including hospital clinicians are not engaging with carers.^10^ In the recently published British Geriatrics Society and Centre for Perioperative Care Guideline for the care of people living with frailty, key recommendations included proactive involvement of carers throughout the patient's journey and prioritising research to understand the patient experience.^3^

To address these knowledge gaps, we investigated older vascular surgery patients' and their carers' experience of care in hospital.

## 2 METHODS

### 2.1 Setting and participants

This study was undertaken at a major metropolitan teaching hospital in New South Wales, Australia. The study recruited patients aged ≥65 years admitted to the vascular surgery unit at Concord Hospital. Where patients had a nominated carer, the carer was also approached for enrollment. Recruitment occurred via multiple methods. Patients and their carers were recruited in‐person towards the end of their hospital stay when they were more likely to be medically stable. If research personnel were not able to meet with the patient and/or carer during the hospital stay, then recruitment occurred via posting the participant information sheet to the patient's address and a follow‐up telephone within a week of discharge from hospital. Carers were defined as the person providing some type of informal, ongoing assistance for the patient. Identification of carers occurred by meeting them in‐person with the patient during the hospital stay or if the patient reported that they had a carer. Consecutive patients admitted under the vascular surgery unit from July 2019 to November 2019 were eligible for inclusion. Inclusion criteria were patients aged ≥65 years and admitted into the vascular surgery unit with an expected length of stay >2 days. Patients, or the person responsible, provided written or verbal informed consent. Patients were excluded if they had cognitive, sensory, or language impairment that was severe enough to impact their ability to complete the study assessments and where there was no‐one to act as their proxy.

### 2.2 Ethics statement

Eligible patients and carers were given a participant information sheet and consent was obtained face‐to‐face prior to hospital discharge or via telephone within a few weeks of returning home. This study was approved by the Sydney Local Health District Human Research Ethics Committee (CH62/6/2019–086).

### 2.3 Study design

We adopted a mixed‐methods convergent design to explore older vascular surgery patients' and carers' experiences of hospital care.^11^ Quantitative methods alone are not adequate to capture the patients' experience of health care and are limited by poor variability and sensitivity, as well as the ceiling effect which results in highly skewed scores.^12^, ^13^ A one‐phase design was used where quantitative and qualitative research strands were collected simultaneously by combining open‐ended questions with rating scales in a questionnaire.^12^, ^14^ Integrative merging analysis of the quantitative and qualitative analyses was undertaken to look for confirmation or discordance between quantitative and qualitative data, and to form more holistic and comprehensive conclusions.^11^, ^15^ The study adhered to Guidelines for consolidated criteria for reporting qualitative research (COREQ) and Guidelines for Checklist of items that should be included in reports of cross‐sectional studies (STROBE).

### 2.4 Study measures and data collection

#### 2.4.1 Patient‐ and carer‐reported experience measures

Patient‐ and carer‐reported experience of their recent hospital admission was explored using a semistructured questionnaire administered via telephone interview one‐month postdischarge from hospital. In the few instances where telephone communication was not possible, for example, due to severe hearing impairment, self‐completion of a postal questionnaire was offered as an alternative. The questionnaire was based on the Leading Better Value Care Inpatient PREM survey, developed by the Ministry of Health's pillar Agency for Clinical Innovation.^16^ In addition to the quantitative measures in this questionnaire, we asked additional open‐ended questions about the best aspects of care and areas for improvement, with probing follow‐up questions included in the interview guide (Appendix 1).

#### 2.4.2 Demographic and health measures

Baseline characteristics of the patients were collected and/or assessed on admission to hospital. These variables included age, sex, place of residence, comorbidity score using the Charlson Comorbidity Index (CCI), mobility status, functional status using the Katz Activities of Daily Living scale, frailty status using the validated 9‐point Clinical Frailty Scale (CFS), cognition using the Abbreviated Mental Test Score (AMTS), admission type (emergency versus elective), and operation details. Cognition and CFS were assessed face‐to‐face on admission by a trained research nurse or doctor. Frail status was defined by a CFS score above 4.^17^ Cognitive impairment was defined by a score below 8 on the AMTS.^18^, ^19^ All other clinical and sociodemographic characteristics of patients were sourced from the medical records. Regarding the carers, their relationship to the patient and their gender was recorded.

### 2.5 Data analysis

Patient characteristics were reported as mean and standard deviation (SD), or median and interquartile range (IQR) for continuous variables, and as frequencies and percentages for categorical variables. Frequencies were used to present quantitative PREM data. All analyses were performed in Microsoft Excel 2013 (Microsoft Inc.).

Qualitative data from the open‐ended patient‐ and carer‐reported experience questions were analysed using content analysis via framework method approach.^20^ Data were compiled in Microsoft Excel. Preliminary deductive codes were based on the quantitative PREMs questions: communication, pain, and medications. This was followed by an in‐depth inductive coding process performed by two investigators. Investigators met to refine codes, develop a working analytical framework, and form final themes. Data were reviewed by a third investigator with experience in qualitative research methods. This review was to ensure no data were overlooked or incorrectly coded and that it was of adequate richness and thickness.^21^ An audit trail of coding decisions was maintained.^22^ Qualitative data were managed using Microsoft Excel and analysed by highlighting words, phrases, and sentences of interest. Domains of data condensation, data display, and conclusion drawing were used to arrive at overarching themes.^23^

Integrative merging analysis of the quantitative and qualitative analyses was undertaken and reported with a contiguous approach, and qualitative data were transformed into numeric counts using content analysis to compare with the quantitative data.^11^, ^15^ This allowed capture of a holistic and complete understanding of the patients' and carers' reported experience of hospital care.

## 3 RESULTS

### 3.1 Participant characteristics

There were 82 patients who met the inclusion criteria of whom forty‐seven (57%) patients were recruited. Nine carers were recruited. Three patients and three carers completed postal questionnaires, while all other patients and carers had the questionnaire administered by a researcher over the telephone. Baseline characteristics of study patients and carers are summarised in Table 1. Mean age was 77 years and 77% were male. Ten (21%) of the patients were frail (CFS score >4), 15 (32%) were dependent in at least one activity of daily living and three (7%) patients had cognitive impairment or dementia. Over half of the patients were electively admitted to hospital and the majority (*n* = 43, 92%) underwent a surgical procedure.

**TABLE 1:** Baseline characteristics of patient (*n* = 47) and carer (*n* = 9) participants.

Characteristic	Data
Patients	
Age (years), mean (SD)	77.3 (8.2)
Male sex, *n* (%)	36 (77)
CALD, *n* (%)	5 (11)
Residential aged care resident, *n* (%)	2 (4)
Living alone^a^, *n* (%)	9 (20)
Receipt of ≥1 formal home support services^a^, *n* (%)	15 (33)
Emergency admission, *n* (%)	14 (30)
Charlson score, median (IQR)	3 (1.0–4.5)
Frail (CFS > 4), *n* (%)	10 (21)
Assisted mobility at admission, *n* (%)	1 (2)
Functional dependence (in ≥1 ADL), *n* (%)	15 (32)
Cognitive impairment (AMTS score <8),^b^ *n* (%)	3 (7)
Underwent operative management, *n* (%)	43 (92)
Underwent major amputation^c^	2 (5)
Carers	
Male sex, *n* (%)	1 (11)
Relationship to patient, *n* (%)	
Spouse—wife	6 (67)
Child–daughter	2 (22)
Child—son	1 (11)

Abbreviations: AMTS, Abbreviated Mental Test Score; ADL, activities of daily living; CFS, Clinical Frailty Scale; CALD, culturally and linguistically diverse; IQR, interquartile range; SD, standard deviation.

^a^ Total living in the community *n* = 45.

^b^ Missing (*n* = 2).

^c^ Major amputation defined as below or above knee amputations or revisions.

### 3.2 Patient‐ and carer‐reported experience of hospital care

Quantitative patient and carer experience data are reported in Table 2. In response to questions about respect, communication, patient engagement, and assessment and management of pain, most patients (74%–89%) responded positively. Among patients who had changes to their medications, 82% reported that they received explanations about these changes. Just over half of the patients felt they received enough information on how to manage at home. For the nine carers, all nine reported being treated with respect and dignity (100%), and most (*n* = 7) reported that their views were heard (78%) and they were kept informed (78%). Comparing the response of the patient and carer for the nine available dyads, similar perceptions were noted on most domains: how well‐informed they felt, whether their views were heard, whether they were given enough information near discharge, whether they felt treated with respect and dignity and the overall rating of care. Differential perceptions were seen in five of the nine dyads regarding whether health professionals explained things in an understandable way.

**TABLE 2:** Summary of quantitative results from patient‐ and carer‐reported experience questionnaire.

Patient experience items	Response, No. (%)			
	Almost/ mostly	Some‐times	Rarely/ Never	Did not apply
I was kept informed as much as I wanted about my treatment and care	39 (83)	4 (9)	4 (9)	
I was involved as much as I wanted in making decisions about my treatment and care	38 (81)	2 (4)	7 (15)	
My views and concerns were listened to	42 (89)	2 (4)	3 (6)	
Did the health professionals explain things in a way you could understand?	37 (79)	7 (15)	3 (6)	
Did you feel you were treated with respect and dignity while you were in the hospital?	42 (89)	4 (9)	1 (2)	
The healthcare professionals involved in my care worked well together as a team	39 (83)	6 (13)	1 (2)	1 (2)
I was asked about my pain levels at least once a day	37 (79)	3 (6)	2 (4)	5 (11)
I received pain relief that met my needs	35 (74)	3 (6)	1 (2)	8 (17)
Changes in my medications were explained to me	27 (57)	5 (11)	1 (2)	14 (30)

	Yes, completely	Yes, to some extent	No, I was not given enough	I did not need this type of information
Do you feel you have been given enough information about how to manage your care at home?	27 (57)	9 (19)	6 (13)	5 (11)

	Very good	Good	Neither good nor poor	Very poor
Overall, how would you rate the care you received while in hospital?	30 (64)	13 (28)	2 (4)	2 (4)

Carer experience items	Almost/mostly	Some‐times	Rarely/Never	Didn't apply
I was kept informed about the treatment and care of the person I look after	7 (78)	1 (11)	0 (0)	1 (11)
I was involved in making decisions about the treatment and care of the person I look after	6 (67)	0 (0)	1 (11)	2 (22)
My views and concerns were listened to	7 (78)	0 (0)	1 (11)	1 (11)
Did the health professionals explain things in a way you could understand?	7 (78)	2 (22)	0 (0)	

	Yes	No		
Were you given specific information to assist you to meet the needs of the person you care for whilst they were in hospital and when they were discharged?	7 (78)	2 (22)		
Did you feel able to look after the person you care for safely/adequately when they went home?	8 (89)	1 (11)		
Did you feel you were treated with respect and dignity while you were in the hospital?	9 (100)	0 (0)		

	Very good	Good	Neither good nor poor	Very poor
Overall, how would you rate the care received by the person you care for?	8 (89)	0 (0)	1 (11)	0 (0)

**FIGURE 1:**
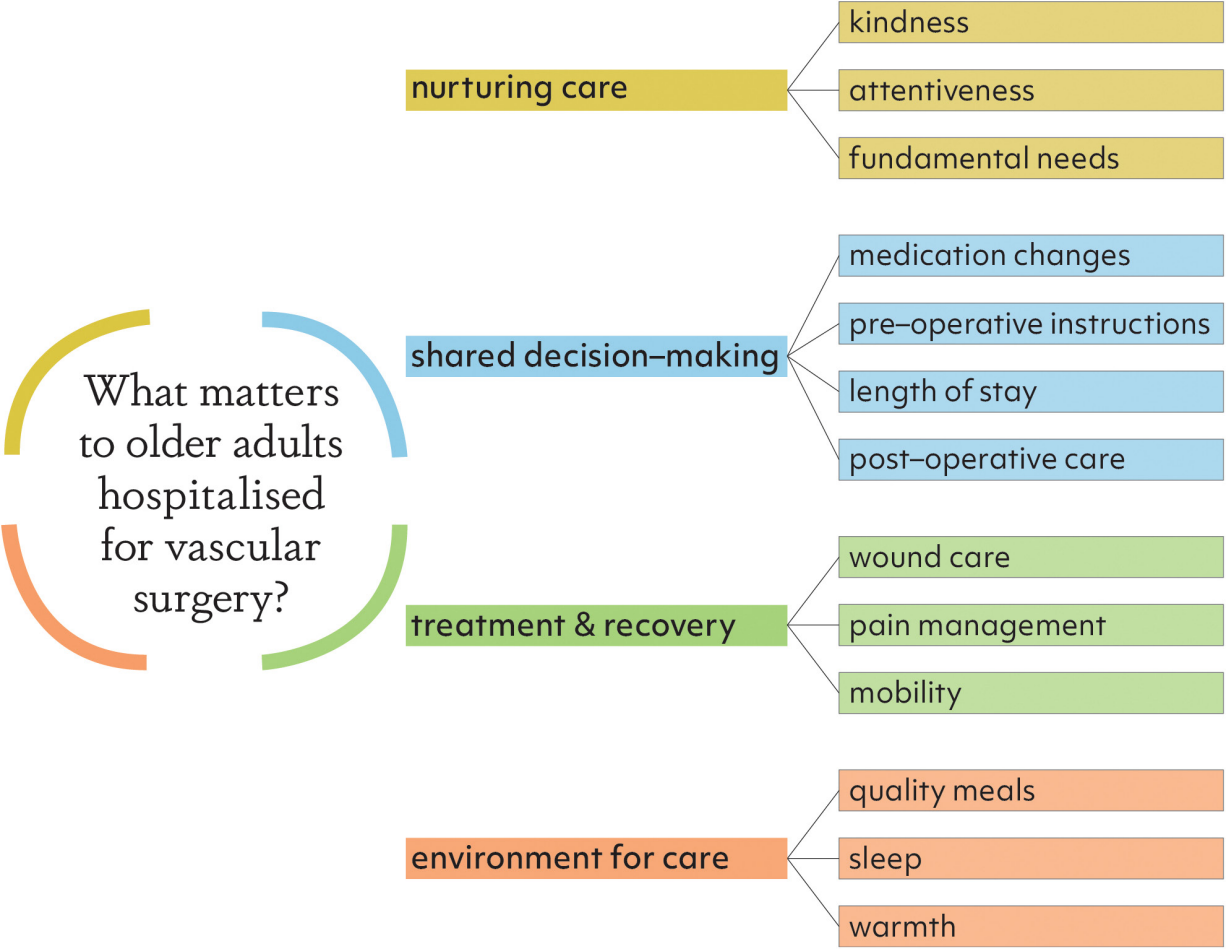
Visual summary of what matters to older vascular surgery patients and their carers based on qualitative content analysis of data.

Qualitative content analysis of the responses to open‐ended questions identified four themes of the patients' and carers' hospital experience (Figure 1).

#### 3.2.1 Theme 1: Nurturing care

Few participants referred to the operation or treatment they received. “Operation” and “theatre” were highlighted by just three patients and two carers. Responses about the best aspects of care related to being “looked after,” made to feel “comfortable” and “treated well.” Comments centred on the clinician's attention to the person's fundamental needs such as food and hygiene:

The hospital care was wonderful; they even opened [food] packets for me as my vision was poor. [female, 94 years, CFS > 4, English‐speaking]

Changing clothes and sheets everyday so he [was] kept clean. [carer, male, 80 years, CFS > 4, Arabic‐speaking]

Participants appreciated the personal attributes of clinicians such as their kindness and attentiveness:

[I] had problems breathing and the nurse came and sat with me, [she] had [her] arm on my shoulder telling me what to do, and everyone was like that! [male, 73 years, CFS < 4, English‐speaking]

These positive comments on the nurturing care received, echoes the quantitative findings, where 42 of the 47 (89%) patients and all nine carers felt they were treated with respect and dignity during their hospital stay.

Negative experiences of care were due to participants perceiving that some clinicians did not have enough time to respond to their needs. Participants valued the availability of clinicians, and the frequency of contact was important. Most participants referred to the care provided by nurses (*n* = 14) and doctors (*n* = 9), while other staff were mentioned less frequently.

#### 3.2.2 Theme 2: Environment for care

The environment for the delivery of care was important to participants. Over a quarter of participants highlighted meals, facilities, and sleeping environment. Many participants struggled with the quality of the meals, with only one patient finding them satisfactory: “I had the best bloody meal!” [male, 89 years, CFS < 4, English‐speaking]. Most participants commented about the lack of variety and flavor of the food. A number of participants reported weight loss in hospital as they found the meals unpalatable. One carer expressed frustration about her family member not receiving the modified meal she required.

Many patients reported difficulty sleeping due to an environment that was busy and noisy at night. Conversely, one participant found that there was an “air of calmness on the ward” [female, 66 years, CFS < 4, English‐speaking].

In response to the question about areas for improvement, issues with the physical environment and equipment were commonly identified by participants. A number reported that the rooms were cold, shared with other patients, cluttered, and that the bathroom facilities were not fit for purpose:

The only thing I didn't like was the “little toilets” [which were a] bit hard to get into. They brought the commode chair which helped. Much easier with the leg and the osteoarthritis. [female, 83 years, CFS > 4, English‐speaking]

Another identified element of the environment for care for patients and carers were measures to reduce the risk of infection:

The infection control was beyond reproach. [female, 66 years, CFS < 4, English‐speaking]

Integrating these qualitative comments with the quantitative data suggests that the concerns about the hospital environment may have accounted for the lower rating of overall care in the hospital by patients.

#### 3.2.3 Theme 3: Shared decision‐making

A range of experiences were described relating to being informed and involved in decision‐making about treatment. While 83% of patients felt they were kept informed always or mostly and 81% felt involved in treatment decisions always or mostly, in the qualitative comments, less than half of patients and carers reported being well‐informed and included when making treatment decisions:

They knew how to talk to me. I could understand. They always asked me when a decision had to be made; like whether to amputate the toe. [male, 90 years, CFS < 4, English‐speaking]

While many indicated that they were consulted and that information was provided, some participants were challenged in understanding this material. Participants were reluctant to ask questions to clarify information:

They spoke slowly and explained [but] I'm not the person to ask so many questions. [male, 74 years, CFS < 4, Spanish‐speaking]

Other patients felt excluded as their family member had been more involved in discussions and decision‐making:

They explained to the wife but not to me. [male, 81 years, CFS < 4, English‐speaking]

Participants particularly felt the lack of consultation and communication occurred with explanations about medications and information relating to pre‐ and postoperative care. Despite 82% of patients who had changes in their medications reporting that they received explanations about these changes in the quantitative measure, some expressed wanting to be better‐informed about their medications in hospital, including new and ceased medications, as well as the indication for the new ones:

Antibiotics as well as blood pressure meds [were] stopped because of my kidneys. When it was stopped, I was not told why …. I could have been better‐informed … nurses told me my meds were changed. It took me most of the day to find a doctor and to explain to me the change in medication. [male, 66 years, CFS < 4, English‐speaking]

Participants also felt that there was an information gap about what to expect before and after surgery. The postoperative course and expected length of stay in hospital were highlighted as being insufficiently communicated:

[I] could have had more info before going in. I thought I would only stay two days and could drive back as I live outside [the state]. They call[ed] it “day surgery”! Then I found out I could not drive four to six weeks [after surgery.] [male, 71 years, CFS < 4]

Patients and carers with diverse cultural and linguistic background (such as Arabic‐ and Spanish‐speaking participants) experienced additional challenges and suggested the use of an interpreter to provide information in the post‐operative period:

Before [the] operation I understood everything but after the operation everything [was] different, for example, the tube for urine …. I think after operation if you have someone who can explain in your language, it would be beautiful. [male, 74 years, CFS < 4, Spanish‐speaking]

#### 3.2.4 Theme 4: Treatment and recovery

While most patients underwent surgery, only a few focused on this as an important part of the experience (*n* = 3 patients, *n* = 2 carers). Those who recounted their experience of surgery felt it was overwhelmingly positive:

[The] operation went beautifully… I did not feel it. I had no after‐effects whatever. [female, 94 years, CFS > 4, English‐speaking]

He had three emergency operations ‐ it was all good. [carer, male, 81 years, CFS < 4, English‐speaking]

There were other aspects of the treatment and recovery that participants did have concerns about. Five patients recounted their pain. Their experiences ranged from satisfaction to frustration, especially about the lack of breakthrough analgesia:

I had to ask for Endone [opioid] for pain, usually it was Panadol [acetaminophen]. Sometimes the pain was excruciating, and I needed this [opioid] every 4 hours! [female, 84 years, CFS < 4, English‐speaking]

Patients described their changed mental and physical function during hospitalisation. One patient recounted his transient memory loss following surgery. Others described their low mood and a need for more social support. Some patients felt they had not received as much physical therapy as they would have liked or required:

[I] needed to be out of bed more. [female, 92 years, CFS > 4, English‐speaking]

Wounds were a concern for many patients and issues relating to the management of their wounds were extensively reported. There were multiple exemplars about pain during dressing changes and issues relating to the continuity of wound care after discharge. A number of participants expressed concerns about the ability of community nurses to provide the complex wound care that they required:

Sometimes community nursing don't know what dressings are required, how to apply [dressings], and [the nurses] don't have them! [male, 66 years, CFS < 4, English‐speaking]

## 4 DISCUSSION

This study's findings about patients and their carer experiences are important in our understanding of hospitalised older surgical cohorts. Older vascular surgery patients and their carers emphasised the importance of receiving personalised care that involved them and went beyond the clinical treatment of their condition. This highlights the need for holistic person‐centred care which is already the driver of recent healthcare system‐wide reforms such as the Age‐Friendly Health System initiative in the United States^24^ and the Acute Frailty Network in the United Kingdom.^25^ Age‐Friendly Health Systems are not currently routinely implemented in Australia but is being trialed by Safer Care Victoria.^26^

As health‐care services move towards developing an Age‐friendly Health System,^24^ an understanding of the patient's experience of care can inform service improvements.^27^ There is a need to expand the routine measurement of patient‐reported measures to surgical services such as vascular surgery where there is a high proportion of older frail patients who are high consumers of health‐care services and most at risk of hospital‐acquired complications. Understanding patient experience was a key recommendation for research in the recently released British Geriatrics Society and Centre for Perioperative Care Guideline for the care of people living with frailty.^3^

To our knowledge, this is the first study to explore patient‐ and carer‐reported experiences of hospital care in older vascular surgery patients. In our study, patients and carers were infrequently concerned about the operation or other treatments received. What was highly valued were their interactions with clinicians and receiving fundamental care. Fundamental care refers to the care activities that are essential for every individual irrespective of their clinical condition or health‐care setting, for example, sleep and meals.^9^ Fundamental care is more likely to be met when there is a trusting relationship between the patient and care provider.^9^ A recent systematic review of qualitative studies that explored older people's experiences in acute care settings concluded that the care experiences in older people were often negative due to feelings of powerlessness and insignificance, and lack of relational care by healthcare staff.^27^ By contrast, we found that the experiences of patients and carers were mostly positive, and this may be due to the good relational care by healthcare staff that was reported. However, meals were often reported as a negative experience, and is an area for improvement. Clinicians need to know what food services patients receive as it is an important part of patients' and carers' hospital experiences.

Over half of our participants indicated that most of the time they received enough information about their treatment and care, including information about changes to medications. However, when discussing areas for improvement, several patients expressed the view that they wanted to be more informed and involved in decisions about their health, in particular about medications and what to expect before and after the operation. Furthermore, in this surgical population, pain management and meeting mental and physical health needs were highlighted as aspects of care that could be improved. Interestingly, these areas of priority for older vascular surgery patients and carers overlap with the “4Ms” of geriatrics care: what matters to the older person, medication, mobility, and mentation.^24^ Our study provides new insights on what matters most for older surgical patients and carers in hospital. The 4Ms form the basis of system‐wide changes being implemented as part of the Institute for Healthcare Improvement–led Age‐Friendly Health System initiative^24^ and is already being applied in the care of older surgical adults, but not yet in Australia.^28^

### 4.1 Strengths and limitations

Strengths of our study include use of an established patient‐reported experience measurement tool. The use of qualitative data allowed a deeper exploration and analysis of patients' and carers' experiences that quantitative data does not permit.^29^ We saw in our study that closed‐ended questions are shallow and often exhibit a positivity bias.^13^ Compared with prior studies, our study cohort was an older population that included individuals with frailty and functional dependence. Very old and frail patients are often underrepresented in clinical studies and therefore the evidence base is not reflective of “real‐world” patients.^1^ We addressed this by using broad recruitment criteria to ensure inclusion of patients from residential care settings and those with frailty or functional dependence. However, those who had severe cognitive or sensory impairment and with no person responsible available were excluded from the study. Only 57% of eligible patients consented to participate in the study, and the number of carers may have been underestimated as recruitment relied partly on the patient nominating a carer. These factors, in addition to this being a single center study, may reduce the generalisability of our findings. Another limitation is that the sample size was not large enough for subgroup analyses to determine differences in patient‐reported experiences by frailty status which is a high‐priority research area in geriatric surgery.^3^, ^5^

## 5 CONCLUSIONS

Our study provides novel data about patient‐reported experiences of hospital care in older vascular surgery patients. Our patients and their carers highlighted the importance of being cared for in a personal and holistic way and being actively informed and involved in all aspects of their treatment. Age‐Friendly Health System initiatives such as the implementation of the 4Ms align well with older patients' and their carers' priorities about the care they receive. Collaborative action is needed to make healthcare system‐wide changes to improve the outcomes and experiences of this cohort which will add further value to the service provided to this important population.

## FUNDING INFORMATION

This work was supported by Sydney Local Health District The Pitch Project and Ageing and Alzheimer's Institute. Dr Thillainadesan was supported by the National Health and Medical Research Council (NHMRC) Medical Research Postgraduate Scholarship and MIGA's Doctors in Training Grants Program.

## CONFLICTS OF INTEREST STATEMENT

Janani Thillainadesan and Vasi Naganathan are Associate Editors of the Australasian Journal on Ageing.

## Data Availability

The data that support the findings of this study are available on request from the corresponding author. The data are not publicly available due to privacy or ethical restrictions.

## APPENDIX 1

### INTERVIEW GUIDE FOR OPEN QUESTIONS IN PREMS QUESTIONNAIRE

#### Patient questionnaire

What were the best aspects of your care?

How can we improve your experience while under vascular surgery at Concord Hospital?

Is there anything else that you would like to tell me about being in hospital?

Is there anything else that you would like to tell me about after you came out of hospital?

*Probing questions—provision of information about condition and treatment including medications, management of pain, involving patient and family/carer, provision of sufficient information prior to discharge, team approach to care*

#### Carer questionnaire

What were the best aspects of the care received in hospital by the person you care for?

How can we improve your experience as the carer when the person you care for is in hospital under vascular surgery?

Is there anything else that you would like to tell me about the time the person you care for was in hospital?

Is there anything else that you would like to tell me about your experience after they got discharged from hospital?

*Probing questions—provision of information about condition and treatment including medications, management of pain, involving patient and family/carer, provision of sufficient information prior to discharge, team approach to care*
